# Research on a Five-Axis Machining Center Worktable with Bionic Honeycomb Lightweight Structure

**DOI:** 10.3390/ma14010074

**Published:** 2020-12-25

**Authors:** Lai Hu, Jun Zha, Fan Kan, Hao Long, Yaolong Chen

**Affiliations:** 1 School of Mechanical Engineering, Xi’an Jiaotong University, Xi’an 710049, China; hulai0405@stu.xjtu.edu.cn (L.H.); jun_zha@xjtu.edu.cn (J.Z.); mlpqwe@stu.xjtu.edu.cn (F.K.); 2 Xi’an Jiaotong University Suzhou Academy, Suzhou 215123, China; 3 Lianyungang Shenying Composite Technology Co. Ltd., Lianyungang 222000, China; ysfoods0512@hotmail.com

**Keywords:** honeycomb structure, CFRP, lightweight workbench, machining efficiency

## Abstract

The processing of high-precision aerospace parts requires not only ultra-precision machine tools, but also high-efficiency processing. However, in order to realize high-efficiency processing, besides optimizing the system and process parameters, some subversive research can also be done on the machine tool structure. In this paper, the lightweight research is mainly carried out on the structure of machine tool worktable. The traditional workbench is very “heavy” and “slowness”. If the traditional workbench is subverted and reformed to reduce the weight, the processing efficiency will be improved qualitatively. Therefore, this paper studies the lightweight worktable of CFRP (carbon fiber reinforced polymer) in combination with the biological “honeycomb” shape. At first, the tensile, bending, compressive and laminar shear analysis of CFRP were carried out, and the comprehensive parameters were obtained. Simultaneously, the theoretical research and the honeycomb structure simulation and verification of CFRP worktable are carried out. The results show that the HACT (honeycomb arrangement of circular tubes) is 18.51% better than the SACT (straight arrangement of circular tubes) and 45.05% better than the OW (original worktable) by comparing and analyzing the weight of the three modes (HACT, SACT and OW). The actual weight of bionic honeycomb lightweight worktable is 1100 kg, while the simulation result is 1080.25 kg, with an error of 1.8%. Meanwhile, it is analyzed that the original workbench weight of the five-axis machining center is 2023 kg, while the simulation result is 1998.6 kg, with an error of 1.2%. The lightweight degree is reduced by 45.05%. However, the actual lightweight degree has been reduced by 45.63%. The error between simulation and actual is less than 1.3%. This kind of structural transformation has brought forward cutting-edge innovations to the machine tool processing industry. It provides a reference scheme for related enterprises in the future equipment renovation.

## 1. Introduction

Every country has attached great importance to the topic of energy conservation. In industry, both researchers and entrepreneurs are very concerned about how to improve the processing efficiency and accuracy of products, while ignoring the saving of raw materials [1]. How can the three issues of energy conservation, processing efficiency and accuracy be balanced? In this paper, the lightweight transformation of the worktable of the aerospace five-axis machine tool is studied. Its purpose is to greatly reduce the loss of raw materials and improve the processing efficiency under the condition of ensuring the processing accuracy. Of course, there are many ways to reduce raw materials and improve processing efficiency and accuracy.

For example, O ‘Driscoll E et al. [2] proposed a new type of non-invasive intelligent energy sensor, which can infer the working state of the machine tool according to the information contained in the power signal recorded at the main input of the machine tool. Therefore, the overall energy efficiency of the manufacturing industry is improved. Apprich S. et al. [3] established a model of vibration avoidance and damping method in order to properly deal with the vibration of the machine tool and improve the processing accuracy and efficiency. Ivan Darío Arango-López et al. [4] proposed two complementary methods. By using the combined grinding of the same machine-traditional polishing and selective polishing processes, higher flatness is realized. This method saves time and energy consumption. Li P Z et al. [5] made a new improvement on the structure of the machine tool by using the topology optimization results. The results show that the flexibility of the optimized structure decreases. The first-third order natural frequencies are improved. The structural quality was reduced by 5.55%. Zulaika J.J. et al. [6] proposed a design method of high efficiency light milling machine in order to reduce the weight and improve the processing efficiency.

The above author considers the optimization of processing parameters, used in order to achieve the improvement of processing efficiency. However, it often ignores the subversive transformation method of the structure. Carbon fiber composite reinforcement material is a very suitable source material for lightweight modification of machine tools. It has obvious advantages of light weight and high strength [7].

For example, in the light weight transformation, Xiang F.F. et al. [8] designed two different stiffened plate structure worktables according to the design requirements of high machining accuracy and light weight of high-speed machining centers, one adopting traditional design method and the other adopting bionic design method. Through static and modal analysis, the superiority of the optimization scheme is verified. Gao D.Q. et al. [9] improved the structure of DVG850 high-speed machining center worktable. The improved worktable maintains the static performance of the original worktable and improves the dynamic performance. However, its mass is 23.2 kg lighter than the original structure. Liu C. et al. [10] put forward an optimization design method based on topology optimization, rib shape selection and arrangement, and size optimization for the weakest link of machine tool column structure. Through the finite element dynamic analysis, modal analysis and harmonious response analysis of the machine tools, it is shown that the column structure is the key component that affects the dynamic performance of the machine tools. L. Kroll et al. [11] put forward the general influence of lightweight design method on the energy efficiency of machine tools and the limitation on the maximum mass reduction of structural components. For different lightweight design methods, a conclusive theoretical research is realized through an example component. Croccolo D. et al. [12] skillfully used lightweight materials, thus eliminating high-consumption hydraulic pump systems. Through the drilling operation on the three-axis CNC unit, the loading input force used for analysis is experimentally evaluated.

Some scholars have also combined carbon fiber composite reinforcement materials to carry out lightweight transformation of machine tools. For example, Frederik Birk et al. [13] proposed an innovative method to use CFRP in hybrid design of machine tool structures, increasing specific stiffness and making it easy to manufacture. Aggogeri Francesco et al. [14] proposed and compared a set of materials that may be excellent candidates for manufacturing machine tool (MT) moving parts. Several prototypes made of aluminum foam sandwich (AFS), aluminum corrugated i sandwich (ACS) and carbon fiber reinforced plastics (CFRP) were evaluated. The results also show that the weight of CFRP structure is reduced by 48.5% while ensuring high rigidity, which can be effectively applied to rough machining and saves MT energy consumption. Khanna, Navneet et al. [15] studied and compared the drilling properties of carbon fiber reinforced plastic (CFRP) composites in dry and low temperature environments. The green processing technology is adopted for cryogenic processing of CFRP composites. Compared with dry drilling, the Ra value of cryogenic drilling is reduced by 14–38%, and the inlet reverse stratification coefficient is increased by 5–68%. These results show the applicability of low temperature drilling to industry. Lee D.G. et al. [16] used adhesives and bolts to connect high modulus carbon fiber epoxy composite sandwiches with welded steel structures to manufacture vertical and horizontal sliders for large numerical control machine tools. On the premise of not sacrificing stiffness, these composite structures reduce the weight of vertical slide rail and horizontal slide rail by 34% and 26% respectively, and increase the damping by 1.5–5.7 times. Bretz A. et al. [17] proposed a method to replace the peripheral parts of traditional machine tools with fiber reinforced plastic structures. A simple and economical production process is adopted to realize rapid manufacturing.

In the above literature, whether from the perspective of improving processing efficiency or saving energy, the final practical goal of lightweight transformation is difficult to apply to practice, and it is also difficult to solve the actual needs of manufacturing enterprises. In order to highlight that lightweight transformation can bring great help to the manufacturing industry. This study mainly carries out lightweight transformation on the worktable of ultra-precision/ultra-high speed five-axis machining center. The bionic honeycomb structure and carbon fiber composite are used for comprehensive innovation research. The comparative analysis of the properties of carbon fiber composites was analyzed, as well as the corresponding models were selected as the material parameters for this study. The carbon tube arrangement and comprehensive factors were also analyzed. Finally, SY-3K carbon fiber composite tube was selected to reform the honeycomb arrangement lightweight worktable. At the same time, the theoretical, simulation and experimental comparison are analyzed. The analysis results improve the lightweight degree of the worktable of the five-axis machining center, improve the processing efficiency and reduce the cost.

## 2. Theoretical Analysis of Five-Axis Machining Center Worktable

Prior to determining which material type and structural arrangement mode to use, it is necessary to carry out structural vibration theoretical analysis on the worktable.

### 2.1. Structural Analysis of Machine Tool Worktable

**Figure 1:**
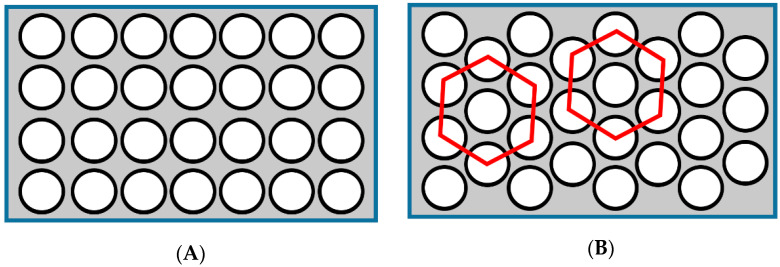
Two modification schemes, (**A**) SACT; (**B**) HACT.

In this study, except the basic analysis of the original structure, two other structures will be analyzed, as shown in Figure 1.

From Figure 1, both arrangement modes are filled with CFRP in the worktable. Figure 1A is the SACT (straight arrangement of circular tubes). Figure 1B is the HACT (honeycomb arrangement of circular tubes).

### 2.2. Vibration Analysis

**Figure 2:**
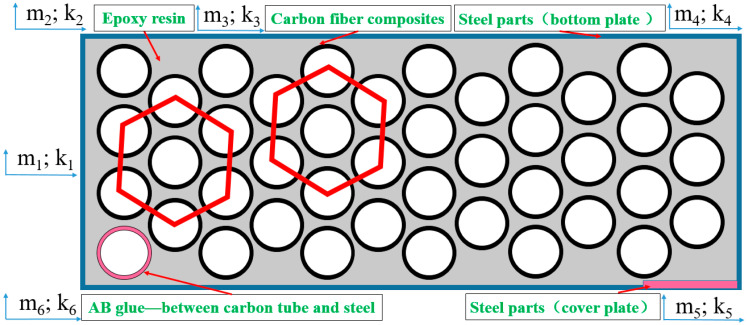
Simplified diagram of lightweight worktable quality for five-axis machining center.

In addition to analyzing the mechanical modification structure of the five-axis machining center worktable, it is also necessary to study the vibration modal stiffness of the machined parts after adding CFRP to the worktable. Therefore, the mass of the lightweight worktable model is simplified through the actual modification of the structure and processing state, as shown in Figure 2.

According to Figure 2, since the first six modes will be analyzed later. So the six-order equation is established. Where the kinetic energy function of the system is [18]:

(1) $$T=\frac{1}{2}(m_{1}{\dot{x}}_{1}{}^{2}+m_{2}{\dot{x}}_{2}{}^{2}+m_{3}{\dot{x}}_{3}{}^{2}+m_{4}{\dot{x}}_{4}{}^{2}+m_{5}{\dot{x}}_{5}{}^{2}+m_{6}{\dot{x}}_{6}{}^{2})$$

The potential energy Equation is:

(2) $$U=\frac{1}{2}[k_{1}x_{1}^{2}+k_{2}{\left(x_{2}-x_{1}\right)}^{2}+k_{3}{\left(x_{3}-x_{2}\right)}^{2}+k_{4}{\left(x_{4}-x_{3}\right)}^{2}+k_{5}{\left(x_{5}-x_{4}\right)}^{2}+k_{6}{\left(x_{6}-x_{5}\right)}^{2}]$$

The energy dissipation function of the system is:

(3) $$D=\frac{1}{2}[c_{1}{\dot{x}}_{1}^{2}+c_{2}{\left({\dot{x}}_{2}-{\dot{x}}_{1}\right)}^{2}+c_{3}{\left({\dot{x}}_{3}-{\dot{x}}_{2}\right)}^{2}+c_{4}{\left({\dot{x}}_{4}-{\dot{x}}_{3}\right)}^{2}+c_{5}{\left({\dot{x}}_{5}-{\dot{x}}_{4}\right)}^{2}+c_{6}{\left({\dot{x}}_{6}-{\dot{x}}_{5}\right)}^{2}]$$

The partial derivatives of the Equations (1)–(3) in the Lagrange equation are obtained, and the differential equations of motion of the lightweight worktable system during machining are obtained by introducing the $m_{1};m_{2};m_{3};m_{4};m_{5};m_{6}$ in Figure 2. The differential equations are expressed by matrices as [17,18],

(4) $$\left[m\right]\left\{\ddot{x}\right\}+\left[c\right]\left\{\dot{x}\right\}+\left[k\right]\left\{x\right\}=\left\{P\right\}$$

where $\left\{x\right\},\left\{\dot{x}\right\},\left\{\ddot{x}\right\},\left\{P\right\},\left[m\right],\left[c\right],\left[k\right]$ represent a displacement array, a velocity array, an acceleration array, an interference force array, a mass matrix, a damping matrix and a stiffness matrix, respectively.

When there is no damping, the Equation (4) can be expressed as:

(5) $$\left[m\right]\left\{\ddot{x}\right\}+\left[k\right]\left\{x\right\}=\left\{0\right\}$$

Let the solution of Equation (5) be:

(6) $$\left\{x\right\}=\left\{A\right\}e^{{iw}_{n^{t}}}$$

Of which, $\left\{A\right\}=\left\{\begin{array}{c} A_{1}\\ A_{1}\\ \vdots \\ A_{n} \end{array}\right\}$, $\left\{x\right\}=\left\{\begin{array}{c} x_{1}\\ x_{2}\\ \vdots \\ x_{n} \end{array}\right\}$.

The first and second derivatives of Equation (6) are obtained respectively, and the main mode equation can be obtained by introducing them into Equation (5) as follows:

(7) $$\left(\left[k\right]-\omega_{n}^{2}\left[m\right]\right)\left\{A\right\}=\left\{0\right\}$$

Obviously, for {A} to have a non-zero solution, the coefficient determinant in Equation (7) must be 0. The frequency equation is obtained:

(8) $$\det\left(\left[k\right]-\omega_{n}^{2}\left[m\right]\right)=\left|\begin{array}{cccc} k_{11}-m_{11}\omega_{n}^{2} & k_{12}-m_{12}\omega_{n}^{2} & \ldots & k_{1n}-m_{1n}\omega_{n}^{2}\\ k_{21}-m_{21}\omega_{n}^{2} & k_{22}-m_{22}\omega_{n}^{2} & \ldots & k_{2n}-m_{2n}\omega_{n}^{2}\\ \vdots & \vdots & \vdots & \vdots \\ k_{n1}-m_{n1}\omega_{n}^{2} & k_{n2}-m_{n2}\omega_{n}^{2} & \ldots & k_{nn}-m_{nn}\omega_{n}^{2} \end{array}\right|$$

The n-th order algebraic equation of $w_{n}{}^{2}$ is obtained by expanding it:

(9) $$\omega_{n}{}^{2n}+a_{1}\omega_{n}{}^{2\left(n-1\right)}+a_{2}\omega_{n}{}^{2\left(n-2\right)}+\cdots +a_{n-1}\omega_{n}{}^{2}+a_{n}=0$$

where $a_{1},a_{2},\cdots ,a_{n}$ is a combination of $k_{ij}$ and $m_{ij}$.

As shown in system Figure 2, for Equation (5) [m] and [k] are: $\left[\begin{array}{cccccc} m_{1} & 0 & 0 & 0 & 0 & 0\\ 0 & m_{2} & 0 & 0 & 0 & 0\\ 0 & 0 & m_{3} & 0 & 0 & 0\\ 0 & 0 & 0 & m_{4} & 0 & 0\\ 0 & 0 & 0 & 0 & m_{5} & 0\\ 0 & 0 & 0 & 0 & 0 & m_{6} \end{array}\right]$ and $\left[\begin{array}{cccccc} k_{1}+k_{2} & -k_{2} & 0 & 0 & 0 & 0\\ -k_{2} & k_{2}+k_{3} & -k_{3} & 0 & 0 & 0\\ 0 & -k_{3} & k_{3}+k_{4} & -k_{4} & 0 & 0\\ 0 & 0 & -k_{4} & k_{4}+k_{5} & -k_{5} & 0\\ 0 & 0 & 0 & -k_{5} & k_{5}+k_{6} & -k_{6}\\ 0 & 0 & 0 & 0 & -k_{6} & k_{6} \end{array}\right]$, respectively. [m] and [k] are introduce into that Equation (7) to obtain:

(10) $$(\left[\begin{array}{cccccc} k_{1}+k_{2} & -k_{2} & 0 & 0 & 0 & 0\\ -k_{2} & k_{2}+k_{3} & -k_{3} & 0 & 0 & 0\\ 0 & -k_{3} & k_{3}+k_{4} & -k_{4} & 0 & 0\\ 0 & 0 & -k_{4} & k_{4}+k_{5} & -k_{5} & 0\\ 0 & 0 & 0 & -k_{5} & k_{5}+k_{6} & -k_{6}\\ 0 & 0 & 0 & 0 & -k_{6} & k_{6} \end{array}\right]-\omega_{n}^{2}\left[\begin{array}{cccccc} m_{1} & 0 & 0 & 0 & 0 & 0\\ 0 & m_{2} & 0 & 0 & 0 & 0\\ 0 & 0 & m_{3} & 0 & 0 & 0\\ 0 & 0 & 0 & m_{4} & 0 & 0\\ 0 & 0 & 0 & 0 & m_{5} & 0\\ 0 & 0 & 0 & 0 & 0 & m_{6} \end{array}\right])\left\{\begin{array}{c} A_{1}\\ A_{2}\\ A_{3}\\ A_{4}\\ A_{5}\\ A_{6} \end{array}\right\}=\left\{\begin{array}{c} 0\\ 0\\ 0\\ 0\\ 0\\ 0 \end{array}\right\}$$

Finally, the six-order main modes expression of the lightweight workbench system during processing is obtained: $\left\{A^{\left(1\right)}\right\},\left\{A^{\left(2\right)}\right\},\left\{A^{\left(3\right)}\right\},\left\{A^{\left(4\right)}\right\},\left\{A^{\left(5\right)}\right\},\left\{A^{\left(6\right)}\right\}$.

## 3. Mechanical Analysis of Carbon Fiber Composites

The manufacturing technology of CFRP is very mature, and there are many models and brands. Therefore, it is particularly important to choose the model and brand suitable for machine tool worktable. This study mainly looks at the differences of different carbon filament quantity combinations and different brands under stretching, bending, compression and layer shear properties, so as to select the best brand and carbon filament quantity.

**Figure 3:**
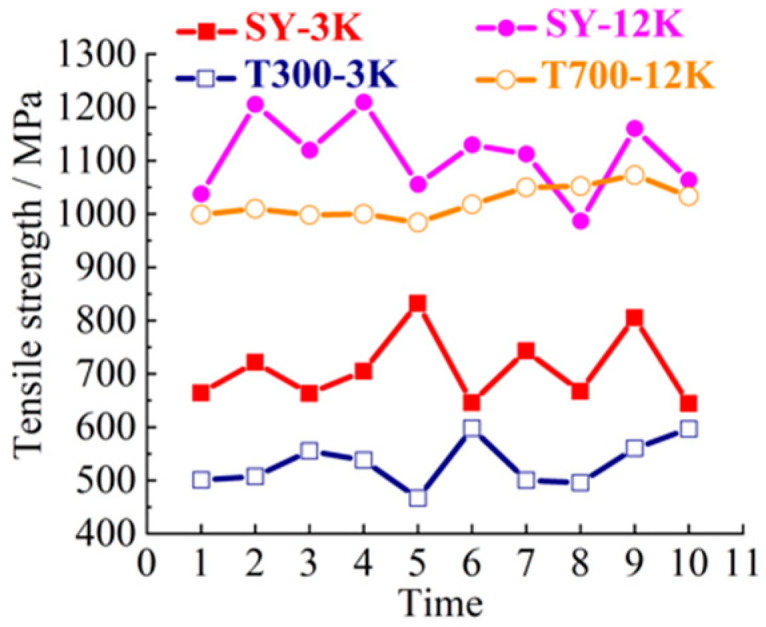
Tensile strength curve.

**Figure 4:**
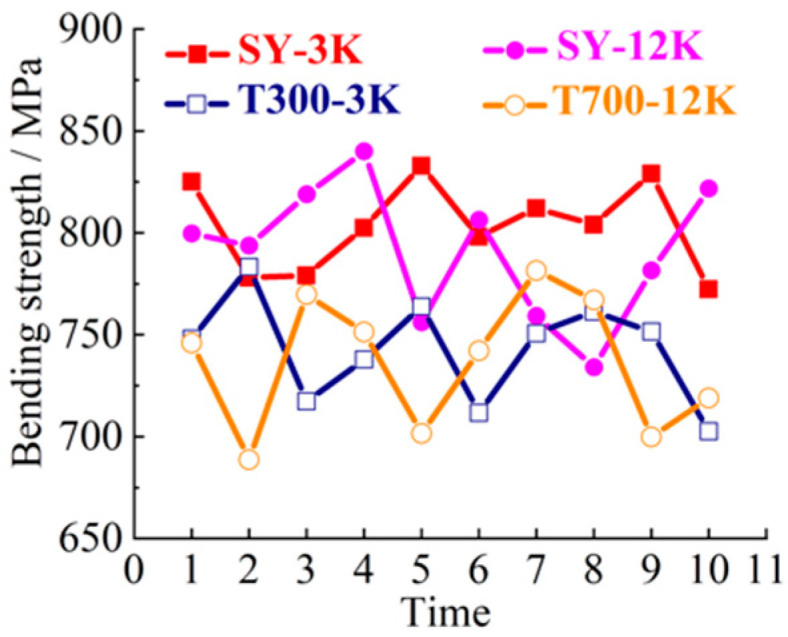
Bending strength curve test.

**Figure 5:**
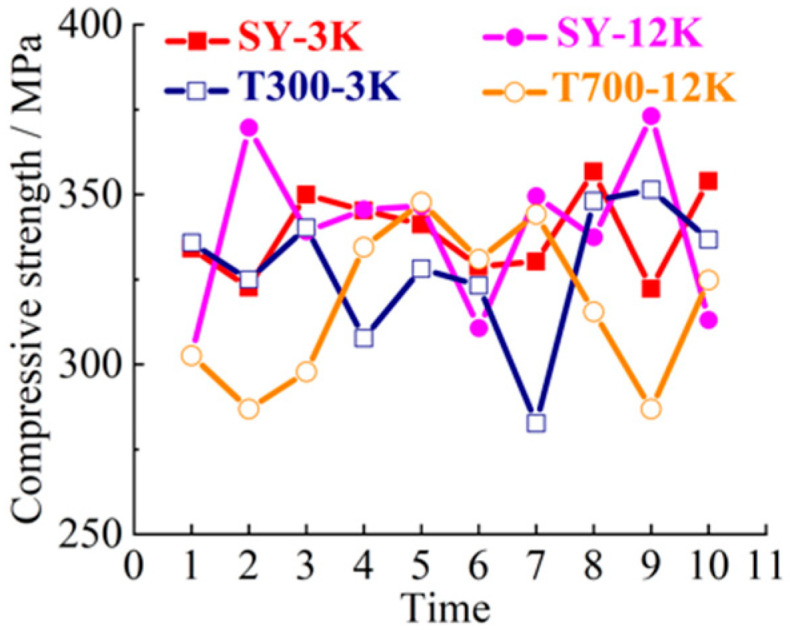
Compression strength curve.

**Figure 6:**
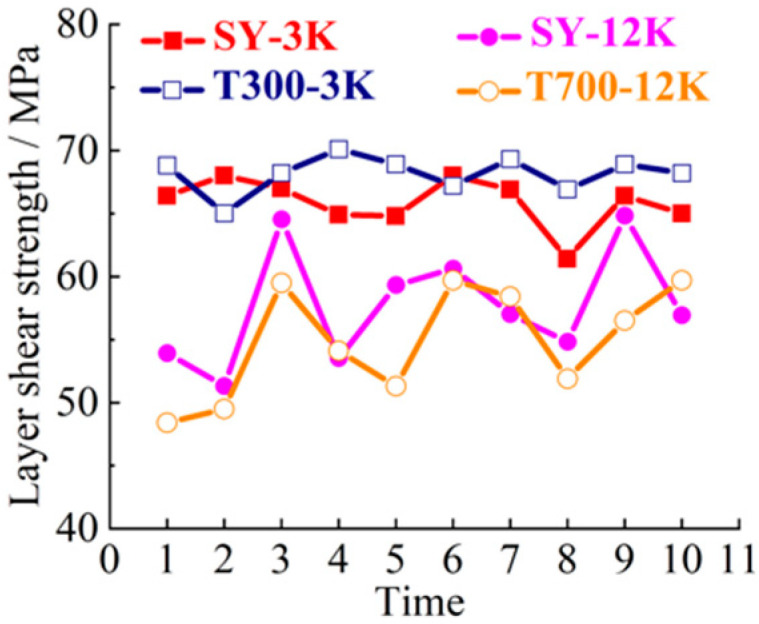
Layer shear strength curve.

SY-3K (Shenying Composite Technology Co.Ltd, Lianyungang, China), T300-3K (Dongli Group, Tokyo, Japan), SY-12K (Shenying Composite Technology Co. Ltd) and T700-12K (Dongli Group) brands and models were selected as the test samples. 3K and 12K represent 3000, and 12,000 carbon filaments, respectively. SY and T represent two brands. The 10 groups of samples were selected for tensile, bending, compression and laminar shear tests. The tensile strength curve is shown in Figure 3. The bending strength curve is shown in Figure 4. The compression strength curve is shown in Figure 5. The layer shear strength curve is shown in Figure 6.

Through 4 different stress tests and analysis results, the comparative test average data and discrete coefficient (CV value) are obtained, as shown in Table 1.

**Figure 7:**
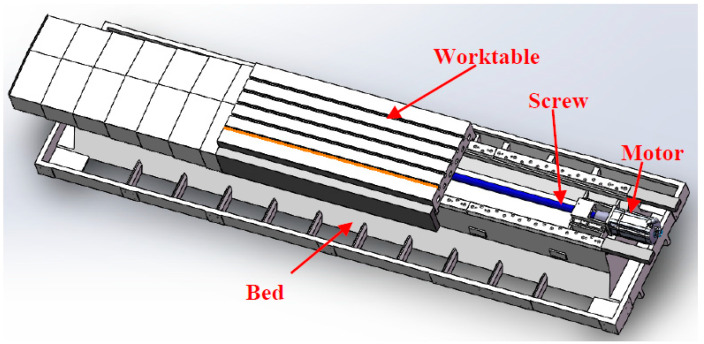
Three-dimensional drawing of horizontal lathe bed of five-axis machining center.

From the macro analysis of Figure 3, Figure 4, Figure 5 and Figure 6, the 10 groups of test samples of T300-3K are the worst in tensile properties. The 10 groups of test samples T700-12K have the worst results in the layer shear performance test. Secondly, since the five-axis machining center in this study belongs to horizontal structure, as shown in Figure 7. Therefore, the worktable is mainly subjected to compression, bending and laminar shear forces. From the Table 1, the highest compressive strength is SY-12K, the highest bending strength is SY-3K, and the highest layer shear strength is T300-3K. Meanwhile, the dispersion coefficient must be less than 6. In the compressive strength, the difference between SY-3K and SY-12K is only 0.18 MPa, while the difference between CV values is 1.5. Considering the cost and comprehensive analysis, SY-3K is selected as the final model for the lightweight transformation of the five-axis machining center worktable in this study.

## 4. Comparative Analysis of Carbon Fiber Composite Honeycomb Structure LightWeight Worktable

According to the previous analysis, the SY-3K model was made into the pipe shape and filled in straight and honeycomb arrangements, but it needs to be simulated before processing.

### 4.1. Comparative Analysis of Honeycomb and Original Structure

**Figure 8:**
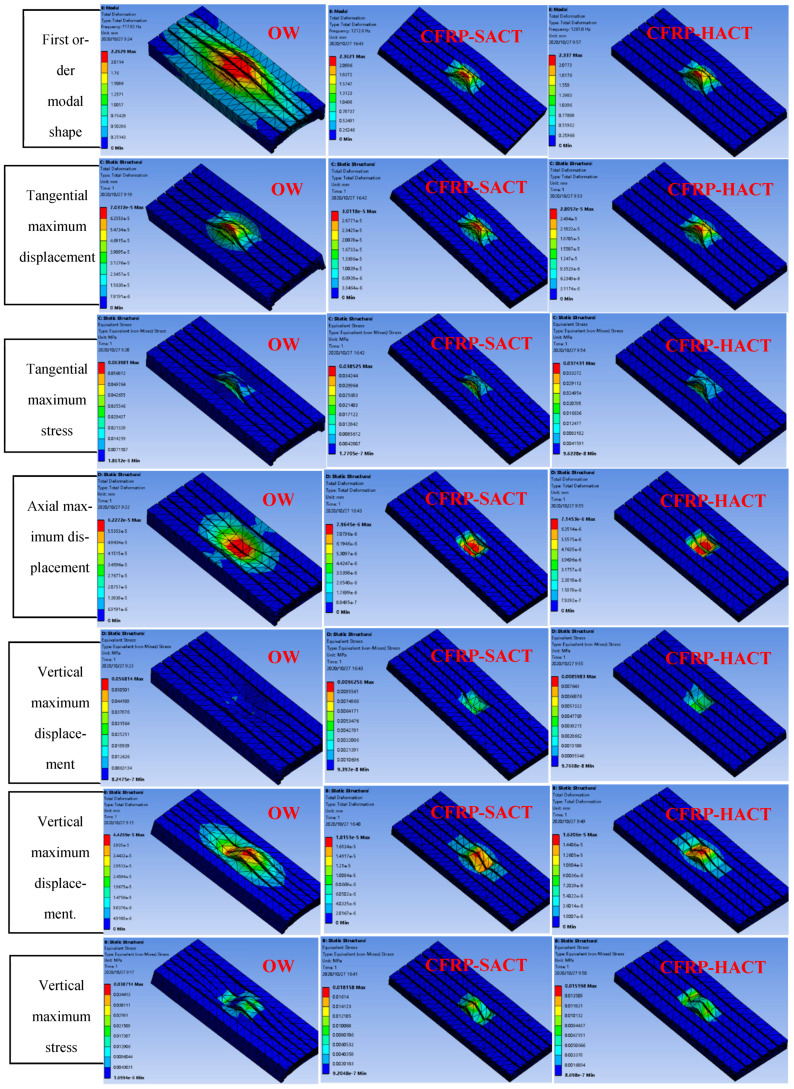
The simulation results.

**Figure 9:**
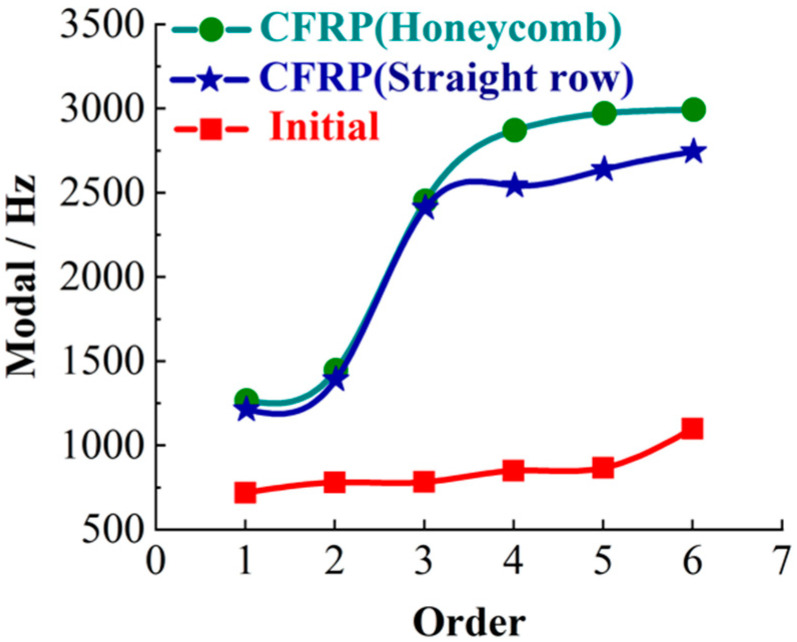
The first six modal curves.

Before simulation, model building, mesh division and boundary conditions need to be applied, as shown in Table 2. Due to the density, Young’s modulus and Poisson’s ratio of epoxy resin need to be considered when filling carbon tubes. The OW, CFRP-HACT and CFRP-SACT are simulated, as shown in Figure 8. In the results, the vibration modes, tangential maximum displacement and tangential maximum stress, axial maximum displacement and axial maximum stress, vertical maximum displacement and vertical maximum stress are mainly analyzed. Finally, the first six modal values (Figure 9) and comparison data (Table 3) are obtained.

From the macroscopic analysis of Figure 8, it can be seen intuitively that the axial, vertical and tangential stresses and deformations of the OW are the largest. According to Figure 9, it can be concluded that the CFRP-HACT has the largest modal. Meanwhile, according to the lightweight results, Table 3 is compared and microscopic analysis shows that except for vertical load deformation, CFRP (whether in straight arrangement or honeycomb arrangement) are superior to the OW (original worktable). The CFRP-HACT is better than the CFRP-SACT. In the vertical load deformation, the CFRP-HACT is slightly lower than that of the OW. However, this does not affect its processing accuracy in any way. Because the force applied in the simulation is the limit value. On the contrary, from the weight comparison and analysis of the three methods, the CFRP-HACT is 18.51% better than the CFRP-SACT and 45.05% better than the OW. Comprehensive analysis of these data shows that when the weight is reduced by 45.05%, the processing efficiency will be greatly increased.

### 4.2. Comparison of Lightweight Indexes between Honeycomb Structure and Original Structure

**Figure 10:**
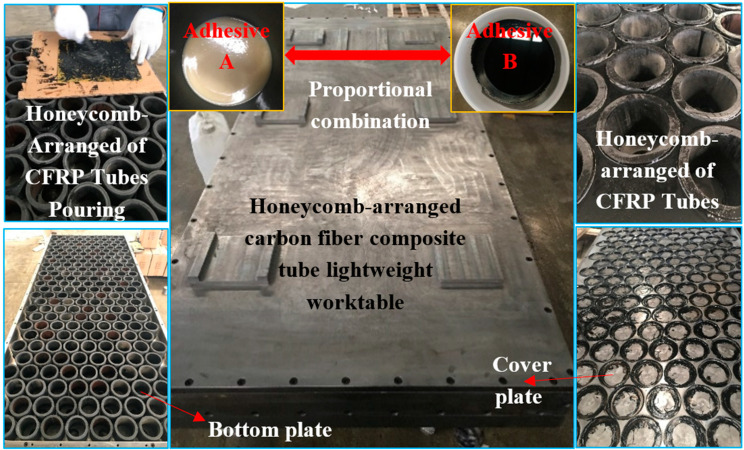
Manufacturing process of honeycomb carbon fiber tube lightweight worktable.

In order to verify the reliability of simulation data, the honeycomb carbon fiber tube lightweight worktable will be manufactured and compared. The manufacturing process is shown in Figure 10.

According to Figure 10, the processing steps of the lightweight worktable are analyzed. Initially, the bottom plate of the worktable is hollowed out, the distributed adhesive AB is applied to one end of the carbon tube, and the bottom plate is bonded. Then, the same proportion of adhesive AB is applied to the cover plate round tube model. Before the cover plate is buckled, epoxy resin shall be filled at the adjacent gaps of the carbon tube on the bottom plate. It is worth noting that the filled epoxy resin must be solidified at normal temperature, which takes about 48 h. Meanwhile, bolts are adopted between the bottom plate and the cover plate. This worktable is connected with 52 bolts. The adoption of such steps is helpful to improve the stability of worktable manufacturing.

**Figure 11:**
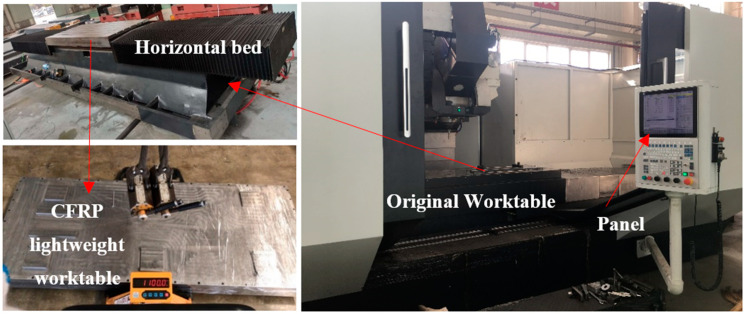
Five-axis machining center honeycomb carbon fiber composite lightweight workbench.

This paper mainly analyzes the lightweight degree of the five-axis machining center worktable. Therefore, lightweight comparison is needed. Assemble the lightweight worktable to the horizontal lathe bed and make comparative analysis, as shown in Figure 11.

According to the comprehensive analysis of Figure 11 and Table 3, the actual weight of the bionic honeycomb lightweight worktable is 1100 kg, while the simulation result is 1080.25 kg, with an error of 1.8%. Likewise, it is analyzed that the original workbench weight of the five-axis machining center is 2023 kg, while the simulation result is 1998.6 kg, with an error of 1.2%. For the Table 3, when analyzing the simulation, the lightweight degree is reduced by 45.05%. However, the actual lightweight degree has been reduced by 45.63%. The error between simulation and actual is less than 1.3%. These results are very reasonable, and provide reliable data support for the lightweight transformation and processing efficiency of the machine tool worktable in the future.

## 5. Conclusions

(1) In this study, the worktable of the five-axis machining center has been subversively lightweight reformed. The worktable of three different structures (OW, HACT and SACT) of the five-axis machining center is analyzed through simulation. In the analysis process, the maximum stress and displacement of the worktable are studied in the axial, vertical and tangential directions, respectively. Similarly, the vibration modes of the worktable are also compared and analyzed. Also, the results are analyzed in detail from macro and micro, and it is found that the CFRP-HACT has the largest modal. Except for vertical load deformation, CFRP (whether in straight arrangement or honeycomb arrangement) are superior to the OW (original worktable). The CFRP-HACT is better than the CFRP-SACT. In the vertical load deformation, the CFRP-HACT is slightly lower than that of the OW. From the weight comparison and analysis of the three methods, the CFRP-HACT is 18.51% better than the CFRP-SACT and 45.05% better than the OW. Comprehensive analysis of these data shows that when the weight is reduced by 45.05%, the processing efficiency will be greatly increased.

(2) The simulation results are compared and analyzed again through experiments, and the actual lightweight degree is reduced by 45.63%, and the error between simulation and reality is less than 1.3%. The results in this study are very reasonable. This is also limited to the square worktable, and most of the force it bears is compression. At the same time, in the analysis of the article, the best structure is the square tube structure. However, this does not affect the research ideas and reliable data that have been provided for the machine tool industry.

## Figures and Tables

**Table 1:** Contrast test data.

Name	Tensile/MPa	CV Value	Bending/MPa	CV Value	Compression/MPa	CV Value	Layer Shear/MPa	CV Value
SY-3K	709.00	9.39	803.25	2.72	338.50	3.75	65.98	3.12
T300-3K	531.58	8.43	742.74	3.44	327.87	6.22	68.15	2.14
SY-12K	1106.22	6.97	790.97	4.22	338.68	7.02	57.66	7.97
T700-12K	1030.00	3.04	736.68	4.43	317.18	7.15	54.90	8.06

**Table 2:** Boundary condition parameters.

Materials	Density (kg/m^3^)	Young’s Modulus (Pa)	Poisson’s Ratio
Carbon fibre	1750	2.1 × 10^11^	0.307
Epoxy resin	980	1.0 × 10^9^	0.38
Structural steel	7850	2.0 × 10^11^	0.3
Gray cast iron	7200	1.1 × 10^11^	0.28

**Table 3:** Comparison of lightweight results.

Mode	**OW**	**CFRP-HACT**	**CFRP-SACT**
Parameters			
Quality (kg)	1998.6	1080.25	1325.5
Axial load-deformation (mm)	4.427 × 10^−5^	1.621 × 10^−5^	1.815 × 10^−5^
Tangential load-deformation (mm)	7.037 × 10^−5^	2.806 × 10^−5^	3.012 × 10^−5^
Vertical load-deformation (mm)	6.227 × 10^−5^	7.145 × 10^−6^	7.965 × 10^−5^
Axial load-stress (MPa)	0.039	0.015	0.018
Tangential load-stress (MPa)	0.064	0.037	0.039
Vertical load-stress (MPa)	0.057	0.009	0.010

## Data Availability

The datasets used or analysed during the current study are available from the corresponding author on reasonable request.
